# An Account of Diseases in an Eastern District of London from October 20, to November 20, 1809

**Published:** 1809-12

**Authors:** 


### *An Account of Diseases in an Eastern District of London from October 20, to November 20, 1809.*

ACUTE DISEASES.

| | |
|---|---|
| Febris Intermittens | 2 |
| Variola | 4 |
| Pneumonia | 3 |
| Pleuritis | 2 |
| Scarlatina | 3 |
| Rheumatismus Acutus | 2 |

CHRONIC DISEASES.

| | |
|---|---|
| Tussis | 25 |
| Dyspnœa | 17 |
| Tussis cum Dyspnœa | 27 |
| Pleurodyne | 6 |
| Hydrothorax | 5 |
| Anasarca | 7 |
| Ascites | 3 |
| Syncope | 2 |
| Hypochondriasis | 5 |
| Leucorrhœa | 3 |
| Menorrhagia | 2 |
| Enterodynia | 5 |
| Diarrhœa | 7 |
| Rheumatismus Chronicus | 10 |

PUERPERAL DISEASES.

| | |
|---|---|
| Dysuria | 3 |
| Menorrhagia Lochialis | 4 |
| Dolores Post Partum | 5 |
| Mastrodynia | 4 |

INFANTILE DISEASES.

| | |
|---|---|
| Convulsio | 3 |
| Diarrhœa | 5 |
| Vermes | 2 |
| Tabes Mesenterica | 2 |

The winter season having now commenced, those diseases which usually make their appearance at this time of the year, begin to engage our attention, and solicit medical aid. Indeed, so little appearance has there been of spring or of summer, and so rapid has been the succession from the close of one winter to the opening of another, that it wears more the appearance of a continuation of one, than the succession of several seasons. In consequence of this, during the whole year, we have had a more than common number of those complaints, which occupy our attention chiefly in the winter quarter. Coughs and catarrhs, peripneumonias and plurisies, however, now begin rapidly to increase, and, owing to the severity of cold, and the prevalence of northerly winds during the last few days, their symptoms have appeared in a more aggravated form.

Numerous instances of the small-pox have lately occurred. For some time past a hope has been entertained, that this disease, formerly so grievous and severe, would, by the substitution of another, have ceased to make its appearance on our list, and indeed would have suffered a total extermination. Our sanguine hopes, however, have received a check.

In a few cases, it has happened that the small-pox has appeared in subjects that have regularly gone through the process of vaccination, and who were supposed hereby to be be secured from the influence of variolous contagion. These facts, which have been but very few, and which when put in the balance against that immense number produced as proofs of the efficacy of the newly discovered means of prevention, are almost light as air, have nevertheless so far increased the prejudices of some, and produced such doubt and hesitation in the minds of others, as to lessen the inclination for using the salutary means of prevention; and the small-pox, either from inoculation, or casual occurrence, is likely to resume its place in the list of diseases.

